# Lyophilized Cell-Free Supernatants of *Lacticaseibacillus paracasei* T0901 Isolated from Fermented Palm Sap Exhibit Antiacne and Antimelanogenic Activities in B16F10 Melanoma Cells

**DOI:** 10.3390/life15121866

**Published:** 2025-12-05

**Authors:** Phoomjai Sornsenee, Nateelak Kooltheat, Nawanwat C. Pattaranggoon, Komwit Surachat, Arnaud Monteil, Chonticha Romyasamit

**Affiliations:** 1 Department of Family and Preventive Medicine, Faculty of Medicine, Prince of Songkla University, Hat Yai 90110, Thailand; ezipnary@gmail.com; 2 Department of Medical Technology, School of Allied Health Sciences, Walailak University, Nakhon Si Thammarat 80160, Thailand; nateelak.ko@wu.ac.th; 3 Faculty of Medical Technology, Rangsit University, Muang Pathumthani, Pathumthani 12000, Thailand; nawanwat.p@rsu.ac.th; 4 Department of Biomedical Sciences and Biomedical Engineering, Faculty of Medicine, Prince of Songkla University, Hat Yai 90110, Thailand; komwit.s@psu.ac.th; 5 Institut de Génomique Fonctionnelle, Université de Montpellier, CNRS, INSERM, 34090 Montpellier, France; arnaud.monteil@igf.cnrs.fr; 6 Department of Physiology, Faculty of Medicine Siriraj Hospital, Mahidol University, Bangkok 10700, Thailand; 7 Research Center in Tropical Pathobiology, Walailak University, Nakhon Si Thammarat 80160, Thailand; 8 Center of Excellence in Innovation of Essential Oil and Bioactive Compounds, Walailak University, Nakhon Si Thammarat 80160, Thailand

**Keywords:** *Lacticaseibacillus paracasei*, postbiotics, antimelanogenic activity, tyrosinase inhibition, CFS

## Abstract

Acne vulgaris is a common chronic inflammatory skin condition. Conventional acne treatments are often limited by adverse effects, driving interest in alternative therapies. This study explored the multifunctional bioactivities of a lyophilized cell-free supernatant (LCFS) derived from *Lacticaseibacillus paracasei* T0901, isolated from fermented palm sap, with a focus on its antimicrobial, antibiofilm, and antimelanogenic potential for dermatological applications. Antimicrobial activity was evaluated using agar well diffusion and broth microdilution assays against acne-associated pathogens, while antibiofilm effects were quantified via crystal violet staining. Antimelanogenic activity was assessed in α-melanocyte-stimulating hormone (α-MSH)-stimulated B16F10 melanoma cells by measuring melanin content and tyrosinase activity. Whole-genome sequencing was performed to identify genes linked to observed bioactivities, and molecular docking was used to predict metabolite–protein interactions. The LCFS exhibited strong inhibitory activity against acne-associated bacteria, with inhibition zones of *C. acnes* (10.67 ± 0.58 mm), *S. epidermidis* (21.00 ± 0.00 mm), and *S. aureus* (20.00 ± 0.00 mm), and a minimum inhibitory concentration of 25 mg/mL. Biofilm formation was significantly reduced by 62.98 ± 3.54%. In α-MSH-stimulated B16F10 cells, LCFS treatment (10 mg/mL) significantly decreased melanin content (73.23 ± 2.36%) and intracellular tyrosinase activity (68.19 ± 6.29%) relative to control. Genomic analysis revealed antioxidant-related genes (*sodA*, *trxB*, *nox*), pigmentation regulators (*mco*, *fcbD*), and *buk* (butyrate kinase), supporting the observed bioactivities. Molecular docking further demonstrated strong binding affinities of LCFS-derived metabolites to tyrosinase and MITF, suggesting modulation of melanogenic pathways. Collectively, these results indicate that *L. paracasei* T0901 produces safe postbiotic compounds with potent antimicrobial, antibiofilm, and antimelanogenic activities, highlighting its promise as a multifunctional ingredient in probiotic-based skincare formulations.

## 1. Introduction

Acne vulgaris is among the most common chronic inflammatory skin disorders worldwide, affecting up to 85% of adolescents and young adults. It is primarily associated with the proliferation of *Cutibacterium acnes*, *Staphylococcus aureus*, *Staphylococcus epidermidis*, and *Malassezia* species [1]. These microorganisms contribute to follicular blockage and inflammation by secreting lipases, proteases, and proinflammatory mediators that disrupt skin homeostasis [1]. Conventional acne treatments, such as topical antibiotics and retinoids, are often limited by adverse effects, including skin irritation, dryness, photosensitivity, and the emergence of antimicrobial resistance [2]. This increasing prevalence of antibiotic-resistant strains has prompted the exploration of alternative therapeutic strategies. Furthermore, the cutaneous inflammation associated with acne lesions frequently stimulates melanocytes, leading to post-inflammatory hyperpigmentation (PIH). This distinct post-inflammatory pigmentary change often persists long after the active infection has been resolved, creating a clinical need for multifunctional agents capable of simultaneously controlling bacterial proliferation and regulating pigmentation.

Melanin, a high-molecular-weight pigment synthesized by melanocytes, determines skin, hair, and eye color and provides photoprotection against ultraviolet (UV) radiation. However, excessive melanin production can lead to hyperpigmentation disorders, such as melasma, freckles, lentigines, and age spots, which can considerably affect the quality of life [3,4]. Melanogenesis is tightly regulated by enzymatic and transcriptional mechanisms involving key proteins such as tyrosinase (TYR), tyrosinase-related proteins 1 and 2 (TRP-1 and TRP-2), and the microphthalmia-associated transcription factor (MITF). MITF functions as a master regulator of melanogenic gene expression; its activation promotes melanin synthesis, whereas its downregulation results in the suppression of pigmentation [5,6]. Clinically, chemical agents such as niacinamide, kojic acid, arbutin, and hydroquinone are commonly employed as tyrosinase inhibitors. However, their potential cytotoxicity, skin irritation, and safety concerns limit their long-term use [7,8,9]. Consequently, there is growing interest in identifying natural and safe antimelanogenic compounds derived from microbial and plant sources [10].

The global skincare market has grown rapidly in recent years, driven by consumer demand for effective, naturally derived products. Consequently, formulations containing probiotics, prebiotics, and postbiotics have attracted attention for their ability to promote skin health by modulating the microbiome [11,12]. This microbiome-friendly cosmetics approach leverages natural bioactives to enhance barrier function, mitigate inflammation, and restore microbial balance. Reflecting the shift toward safe and biocompatible alternatives, probiotic-based skincare is among the fastest-growing segments [13]. Specifically, the global probiotic skincare ingredients market is projected to reach USD 982.3 million in 2025 and USD 3151.6 million by 2035, representing a 221% increase with a compound annual growth rate of 12.4% over the decade [14].

Probiotics are defined as live microorganisms that, when administered in adequate amounts, confer health benefits to the host [15]. They are increasingly considered alternative agents that promote overall health and aid in disease prevention. Some probiotic strains, including *Bifidobacterium*, *Saccharomyces*, *Enterococcus*, *Bacillus*, and *Lactobacillus*, have been extensively documented for their beneficial effects on human health [16]. Initially recognized for maintaining gastrointestinal balance, probiotics are now known to exert positive effects on extraintestinal organs, including the skin [17]. Members of the lactic acid bacteria (LAB) group, particularly the genus *Lacticaseibacillus* (formerly *Lactobacillus*), are among the most extensively studied probiotics, owing to their diverse biological activities, including anti-inflammatory [18], antioxidant [18], and immunomodulatory effects [19]. LAB-derived substances enhance skin barrier function, reduce oxidative stress, and alleviate symptoms of acne and atopic dermatitis [20]. Moreover, cell-free supernatants (CFSs) and cell lysates derived from beneficial bacteria contain metabolites, peptides, and biosurfactants with potent antimicrobial and anti-inflammatory properties that support skin health [21,22]. Recently, probiotics have gained attention as natural and safe alternatives in cosmetics, demonstrating the ability to inhibit acne-associated bacteria through the production of organic acids, bacteriocins, and biosurfactants [23,24]. Specifically, *L. plantarum*-GMNL6 can inhibit *S. aureus* and *C. acnes* [25], while *L. plantarum* APsulloc 331261 and *L. plantarum* APsulloc 331266 can inhibit five skin pathogenic strains—*S. aureus*, *C. acnes*, *Candida albicans*, *Malassezia globose*, and *Malassezia restricta* [26].

Beyond their antimicrobial potential, probiotics and postbiotics have been increasingly recognized as effective antimelanogenic agents in other cosmetic and dermatological applications [11]. For example, metabolites secreted by probiotic strains have demonstrated potential in suppressing melanogenesis by inhibiting tyrosinase activity and downregulating the expression of MITF and other melanogenesis-related genes. In particular, the CFS of *Lactobacillus fermentum* JNU532 reduces tyrosinase activity by 16.7% and melanin content by 23.2% in B16F10 cells [27]. The CFS of *Lactobacillus gasseri* BNR17 exhibits antimelanogenic and antioxidative effects in B16F10 melanoma and HaCaT keratinocyte cells [28]. Moreover, fermented plant-based substrates serve as valuable sources of beneficial LAB strains with significant potential for cosmetic and pharmaceutical applications. Fermented palm sap, a traditional Thai beverage of cultural importance, harbors diverse microbial communities, including various *Lacticaseibacillus* species that are uniquely adapted to high-sugar, low-pH environments. These environmental pressures promote the selection for strains capable of producing stable and bioactive metabolites with potential therapeutic applications [29]. Notably, we have previously isolated *Lacticaseibacillus paracasei* T0901 from fermented palm sap and identified it as a promising probiotic candidate with notable antibacterial activity and metabolite stability [18,29]. However, the antiacne and antimelanogenic potential of the CFS of *L. paracasei* T0901 remains unknown.

Therefore, in the present study, we aimed to investigate the antiacne and antimelanogenic activities of lyophilized CSF (LCFS) derived from *L. paracasei* T0901. To this end, we assessed the antimicrobial activity of the LCFS against acne-associated pathogens, including *C. acnes*, *S. aureus*, and *S. epidermidis*, and evaluated its inhibitory effects on melanin synthesis in α-melanocyte-stimulating hormone (α-MSH)-stimulated B16F10 melanoma cells. Finally, we investigated the potential genetic mechanisms underlying the antimelanogenic effects of the LCFS by analyzing the expression of genes associated with tyrosinase activity using whole-genome sequencing (WGS).

## 2. Materials and Methods

### 2.1. Bacterial Strains and Culture Conditions

*Lacticaseibacillus paracasei* T0901, previously isolated from fermented palm sap and characterized as a potential probiotic [29], was cultured in de Man, Rogosa, and Sharpe (MRS) broth (HiMedia, Mumbai, India) at 37 °C for 24 h. The isolate was stored at −80 °C in 30% (*v*/*v*) glycerol (Sigma-Aldrich, St. Louis, MO, USA) until further use.

*Cutibacterium acnes* ATCC 6919 was obtained from the American Type Culture Collection (ATCC) (Manassas, VA, USA), maintained on blood agar plates, and incubated under anaerobic conditions at 37 °C for 48 h. A single colony was subsequently inoculated into 20 mL of Brain Heart Infusion (BHI) broth and cultured anaerobically at 37 °C for 48 h. Following incubation, bacterial stocks were prepared in 30% (*v*/*v*) glycerol and stored at −80 °C until use.

Both *S. aureus* DMST 4745 and *S. epidermidis* DMST 15548 were cultured in BHI broth (HiMedia) at 37 °C for 24 h. Isolates were stored at −80 °C in 30% (*v*/*v*) glycerol (Sigma-Aldrich) until further use.

### 2.2. Preparation of CFS

CFSs were prepared as described by Sornsenee et al. [18], with minor modifications. Briefly, *L. paracasei* T0901 was inoculated into 200 mL of MRS broth and incubated at 37 °C for 18 h under anaerobic conditions. The culture was centrifuged at 10,000× *g* for 10 min at 4 °C to remove bacterial cells. The supernatant was subsequently passed through a sterile 0.22 µm pore-size filter unit (Sigma-Aldrich) to obtain a cell-free fraction. The filtrate was collected and lyophilized for further experimental use.

### 2.3. Lyophilization

The CFS of *L. paracasei* T0901 was first frozen at −80 °C for 24 h, and the frozen samples were then lyophilized using a freeze-dryer (Lyophilization Systems, Inc., Kingston, NY, USA) under controlled conditions (temperature: −40 °C to −30 °C; pressure: 0.2 mbar). The entire freeze-drying process was completed within 24 h. The lyophilized powders were stored at −20 °C until further use.

### 2.4. Agar Well-Diffusion Assay

This assay was conducted as described by Romyasamit et al. [30] with minor modifications. Briefly, the LCFS of *L. paracasei* T0901 was rehydrated in sterile deionized water to a concentration of 100 mg/mL. Suspensions (1 × 10^5^ CFU/mL) of pathogenic bacteria (*C. acnes* ATCC 6919, *S. aureus* DMST 4745, and *S. epidermidis* DMST 15548) were spread onto blood agar plates supplemented with 5% (*v*/*v*) defibrinated blood. Wells were aseptically cut into the agar using sterile Pasteur pipettes, and 50 μL of LCFS was added to each well. The inoculated plates were incubated under anaerobic conditions at 37 °C for 48 h. Antibacterial activity was assessed by measuring the diameter of the inhibition zones (mm) surrounding the wells. Each experiment was performed in triplicate, and the results are expressed as the mean inhibition zone diameters.

### 2.5. Minimal Inhibitory Concentration (MIC) and Minimal Bactericidal Concentration (MBC) Assays

The MICs and MBC of the LCFS derived from *L. paracasei* T0901 against *C. acnes* ATCC 6919, *S. aureus* DMST 4745, and *S. epidermidis* DMST 15548 were determined as previously described, with minor modifications [31]. The assays were performed using a broth microdilution in sterile 96-well microplates. LCFS was serially twofold diluted in sterile distilled water, ranging from 50 mg/mL to 0.78 mg/mL. Erythromycin (0.0156–8 μg/mL) was used as the positive control. Suspensions were adjusted to approximately 1 × 10^5^ CFU/mL in BHI broth, and 200 μL aliquots containing various concentrations of LCFS or erythromycin were inoculated into wells. Plates were then incubated anaerobically at 37 °C for 48 h. The MIC was defined as the lowest concentration of LCFS that completely inhibited visible bacterial growth.

To determine the MBC, 10 μL samples from wells exhibiting no visible growth were spread onto BHI agar plates and incubated under the same conditions. The MBC was recorded as the lowest concentration resulting in complete bacterial growth inhibition. All experiments were conducted in triplicate.

### 2.6. Biofilm Inhibition Assay

The effect of LCFS from *L. paracasei* T0901 on biofilm formation by *C. acnes* ATCC 6919, *S. aureus* DMST 4745, and *S. epidermidis* DMST 15548 was evaluated as previously described [32], with slight modifications. Overnight cultures of each pathogen were adjusted to a cell density of 5 × 10^5^ CFU/mL in Mueller–Hinton broth and inoculated into 96-well plates containing LCFS at 0.5×, 1×, and 2× MIC. Following incubation at 37 °C for 24 h, planktonic cells were removed, and the wells were washed three times with phosphate-buffered saline (PBS). The biofilms were fixed with 99% methanol for 15 min, stained with 0.1% crystal violet for 10 min, and washed four times with distilled water. The dye was solubilized with 95% ethanol, and absorbance (optical density [OD]) was measured at 570 nm. All assays were performed in triplicate. Biofilm inhibition (%) was calculated as:

Biofilm inhibition (%) = [(OD 570 of control well − OD 570 of treated well)/OD 570 of control well] × 100.

### 2.7. Biofilm Eradication Assays

The ability of the LCFS derived from *L. paracasei* T0901 to eradicate preformed biofilms of *C. acnes* ATCC 6919, *S. aureus* DMST 4745, and *S. epidermidis* DMST 15548 was evaluated following the method described by Perumal et al. [33] with some modifications. Overnight cultures of each pathogen were added to 96-well plates and incubated at 37 °C for 48 h to allow biofilm formation. The wells were then rinsed with PBS to remove non-adherent cells. Established biofilms were treated with LCFS at 0.5×, 1×, and 2× MIC and incubated at 37 °C for 24 h. Following incubation, the wells were washed three times with PBS and stained with 0.1% (*w*/*v*) crystal violet as described above. Absorbance was measured at 570 nm, and all assays were conducted in triplicate. Biofilm eradication (%) was calculated using the following equation:

Biofilm eradication (%) = [(OD 570 of control well − OD 570 of treated well)/OD 570 of control well] × 100.

### 2.8. Tyrosinase Inhibition Assay

The inhibitory effect of the LCFS derived from *L. paracasei* T0901 on mushroom tyrosinase activity was evaluated in a cell-free system. Briefly, each well of a 96-well microplate contained 20 μL of LCFS at final concentrations ranging from 5 to 10 mg/mL, 10 μL of tyrosinase enzyme (2000 U/mL), and 230 μL of PBS (100 mM, pH 6.8). Kojic acid at final concentrations of 1 and 0.5 mg/mL served as the positive control. The reaction mixture was pre-incubated at 25 °C for 10 min, after which 40 μL of L-DOPA (1.5 mM) was added as the substrate. Plates were incubated at 37 °C for 1 h, and the formation of dopachrome was measured at OD 475 nm using a microplate reader. All experiments were conducted in triplicate. The percentage of tyrosinase inhibition was calculated using the following formula:

Tyrosinase inhibition (%) =  (A_control_ − A_sample_)/A_control_ × 100

### 2.9. Cell Viability Assay

The cytotoxicity of LCFS derived from *L. paracasei* T0901 against B16F10 melanoma cells was evaluated using the MTT assay as previously described [34], with some modifications. Briefly, B16F10 cells were seeded in 96-well plates at a density of 5 × 10^3^ cells/well and incubated at 37 °C in a humidified atmosphere containing 5% CO_2_ for 24 h. Cells were then treated with LCFS (0.31 mg/mL to 10 mg/mL) for 24 h. Subsequently, the culture medium was removed, and cells were gently washed once with PBS. MTT solution (0.5 mg/mL) was added to each well, and plates were incubated for 2 h at 37 °C. The medium was removed, and 200 μL of dimethyl sulfoxide (DMSO) was added, and absorbance was then measured at 570 nm using a microplate reader. Cell viability was expressed as a percentage relative to the untreated control group. All experiments were conducted in triplicate.

### 2.10. Trypan Blue Dye Exclusion Assay

Cell viability was further confirmed using the trypan blue dye exclusion method [35]. Briefly, B16F10 melanoma cells were seeded at a density of 5 × 10^3^ cells/well in 96-well plates and incubated for 24 h at 37 °C in a humidified atmosphere containing 5% CO_2_. Cells were then stimulated with α-MSH (1 μM; Sigma-Aldrich) and treated with the LCFS of *L. paracasei* T0901 at concentrations ranging from 0.31 to 10 mg/mL or with kojic acid (0.5–1 mg/mL) as a positive control. After 48 h of treatment, cells were washed twice with PBS and detached using 0.25% trypsin–EDTA. Harvested cells were centrifuged, and the pellets were resuspended in 100 μL of PBS. For staining, 20 μL of the suspension was mixed with 20 μL of 0.4% trypan blue and incubated for 2 min at room temperature. The mixture was loaded onto a hemocytometer, and viable and non-viable cells were counted under a light microscope. Cell viability was calculated as the percentage of live treated cells to live control cells. All experiments were conducted in triplicate.

### 2.11. Measurement of Melanin Content

The effects of LCFS on melanin production were evaluated in B16F10 melanoma cells [27]. Cells were seeded in 96-well plates at a density of 1 × 10^5^ cells/well and incubated at 37 °C in a humidified atmosphere containing 5% CO_2_ for 24 h. The cells were then stimulated with α-MSH (1 μM; Sigma-Aldrich) and treated with LCFS at concentrations ranging from 0.31 to 10 mg/mL or kojic acid (1000 μg/mL) as a positive control for 48 h. After treatment, the cells were washed twice with PBS and harvested using trypsinization. Cell suspensions were centrifuged at 15,000× *g* for 10 min and then dissolved in 1 N NaOH at 70 °C for 1 h. Melanin content was measured at 475 nm using a microplate reader and expressed as a percentage of the α-MSH-stimulated control, which was set at 100%. All experiments were conducted in triplicate.

### 2.12. Cellular Tyrosinase Activity Assay

The effects of the LCFS derived from *L. paracasei* T0901 on intracellular tyrosinase activity were determined in B16F10 melanoma cells using a previously described method [36], with slight modifications. Cells were seeded in 96-well plates at a density of 5 × 10^3^ cells/well and incubated at 37 °C in a humidified 5% CO_2_ atmosphere for 24 h. After incubation, cells were stimulated with α-MSH (1 μM; Sigma-Aldrich) and treated with LCFS (0.31–10 mg/mL) or kojic acid (1000 μg/mL; positive control) for 48 h. Subsequently, the cells were washed with cold PBS and lysed with 1% Triton X-100 in PBS. Lysates were then centrifuged at 12,000× *g* for 10 min at 4 °C to obtain supernatants. Protein concentrations were normalized to 40 μg per reaction using 0.1 M sodium phosphate buffer. Each reaction mixture consisted of 50 μL of lysate and 50 μL of 2.5 mM L-DOPA in a 96-well plate. After incubation at 37 °C for 1 h, dopachrome formation was quantified at 475 nm using a microplate reader. Tyrosinase activity was expressed relative to the α-MSH-treated control, which was set at 100%. All experiments were performed in triplicate.

### 2.13. WGS and Functional Gene Annotation Analysis

Genomic DNA of *L. paracasei* T0901 was extracted and purified using the DNeasy Extraction Kit (QIAGEN, Hilden, Germany) according to the manufacturer’s protocol. Briefly, bacterial cells were suspended in 180 μL of lysis buffer and incubated at 37 °C for 30 min, followed by the addition of 25 μL of proteinase K and 200 μL of buffer AL, and further incubation at 56 °C for 30 min. Subsequently, 200 μL of ethanol was added, and the mixture was centrifuged at 5000× *g* for 1 min. DNA was washed with 500 μL of buffer AW2 and eluted using buffer AE. DNA purity and concentration were evaluated spectrophotometrically at 260/280 nm and confirmed using agarose gel electrophoresis (Sigma-Aldrich).

Purified genomic DNA was subjected to sequencing using the Illumina NextSeq^TM^ 550 platform (Illumina Inc., San Diego, CA, USA). Raw paired-end reads (150 bp) were quality-filtered, and low-quality reads were trimmed. Genome assembly, annotation, and quality assessment were performed using the BacSeq v1.0 pipeline. Genes encoding putative bacteriocins were identified through sequence similarity searches and visualized using the BAGEL4 webserver [37]. Functional annotation for tyrosinase- and antioxidant-related genes was conducted using UniProt online database (UniProt Consortium; https://www.uniprot.org/).

### 2.14. Molecular Docking

#### 2.14.1. Generation of 3D Structural Models

The 3D structures for both the receptor, human tyrosinase (UniProt: P14679), and the seven microbial ligands were determined using AlphaFold prediction models [38,39]. The Tyrosinase structure was sourced from the AlphaFold Protein Structure Database. The ligand proteins, which included Enterocin X, Carnocin CP52, LSEI 2386, Thermophilin A, Multicopper oxidase, Putative butyrate kinase, and Gamma-glutamyl-gamma-aminobutyrate hydrolase, were also modeled using AlphaFold. These structural models were then prepared for docking.

#### 2.14.2. Protein–Protein Docking Simulation

Molecular docking simulations were performed to predict how the microbial ligands bind to the human tyrosinase receptor. Docking was carried out using the HDOCK SERVER (http://hdock.phys.hust.edu.cn), an online tool that uses a hybrid method of template-based modeling and ab initio docking for protein interactions [40]. The human tyrosinase acted as the receptor against all seven AlphaFold-predicted ligand structures. For detailed analysis, the ten best docking results from each simulation were examined. Among these, the complex with the lowest (most favorable) docking score and a suitable position near the catalytic site of human tyrosinase was chosen for further interaction analysis.

#### 2.14.3. Interaction Analysis

The detailed binding characteristics, including non-covalent contacts, of the predicted complexes were analyzed using the Protein–Ligand Interaction Profiler (PLIP) tool (https://plip-tool.biotec.tu-dresden.de) [41]. PLIP automatically identified interaction types such as hydrophobic contacts, hydrogen bonds, salt bridges, and other interactions. Note that the specific criteria for defining hydrogen bonds were a maximum donor–acceptor distance of 4.1 Å and a minimum donor–H–acceptor angle of 100°. Hydrophobic interactions were confirmed by checking close carbon–carbon atom distances within a predefined threshold.

#### 2.14.4. Statistical Analysis

All data are presented as mean ± standard deviation (SD) from at least three independent experiments. Statistical significance between groups was assessed using one-way analysis of variance (ANOVA), followed by post hoc testing where appropriate, or by Student’s *t*-test for pairwise comparisons. A *p*-value < 0.05 was considered statistically significant.

## 3. Results

### 3.1. LCFS Derived from L. paracasei T0901 Exhibits Antimicrobial Activity Against the Tested Acne-Associated Bacteria

The LCFS derived from *L. paracasei* T0901 exhibited potent inhibitory activity against acne-associated bacteria, with inhibition zones against *C. acnes* ATCC 6919 (10.67 ± 0.58 mm), *S. epidermidis* DMST 15548 (20.33 ± 0.47 mm), and *S. aureus* DMST 4745 (21.00 ± 0.00 mm). The strongest inhibition was observed against *S. aureus*, followed by *S. epidermidis* and *C. acnes*.

We then evaluated the antibacterial activity of the LCFS of *L. paracasei* T0901 against *C. acnes* ATCC 6919, *S. epidermidis* DMST 15548, and *S. aureus* DMST 4745 using a broth microdilution assay. As presented in Table 1, the LCFS of *L. paracasei* T0901 exhibited strong antibacterial activity, inhibiting all three pathogenic bacteria with MIC values of 25 mg/mL. The MBC values for all tested strains exceeded 50 mg/mL. These results indicate that LCFS can inhibit the growth and activity of acne-associated bacteria.

### 3.2. LCFS from *L. paracasei* T0901 Inhibits Biofilm Formation and Eradicates Established Biofilms of Acne-Associated Bacteria

**Figure 1:**
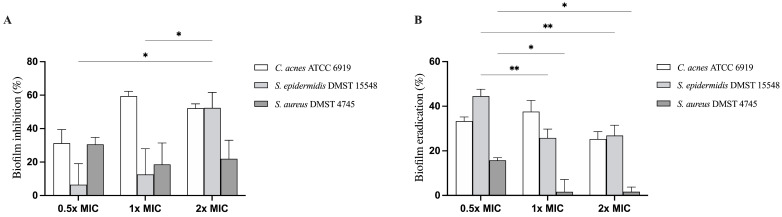
Effects of the lyophilized cell-free supernatant of *Lacticaseibacillus paracasei* *T0901* on the inhibition of biofilm formation (**A**) and eradication of established biofilms (**B**). The percentage inhibition of each data point was calculated relative to its negative control. Data are presented as mean ± standard deviation (* significant difference; * *p* < 0.05, ** *p* < 0.01).

We assessed the inhibitory activity of the LCFS derived from *L. paracasei* T0901 against biofilm formation by the tested strains using the crystal violet assay. As depicted in Figure 1A, treatments with the LCFS significantly reduced biofilm formation in a dose-dependent manner for all tested bacteria. At 1× MIC, biofilm inhibition reached 59.42 ± 2.86% for *C. acnes* ATCC 6919, 12.65 ± 15.34% for *S. epidermidis* DMST 15548, and 18.63 ± 12.82% for *S. aureus* DMST 4745. At 2× MIC, the inhibitory effect of the LCFS on biofilm formation remained high for *C. acnes* (52.18 ± 2.66%) and increased in *S. epidermidis* (52.39 ± 9.26%) and *S. aureus* (21.96 ± 11.08%). The highest biofilm inhibition was observed against *C. acnes* at 1× MIC and *S. epidermidis* at 2× MIC. These results indicate that LCFS can disrupt the ability of acne-associated bacteria to form biofilms.

We assessed the ability of the LCFS derived from *Lacticaseibacillus paracasei* T0901 to eradicate preformed biofilms using the crystal violet assay. As presented in Figure 1B, treatment with the LCFS resulted in a significant reduction in biofilm biomass of all tested strains in a dose-dependent manner (*p* < 0.05). At 1× MIC, biofilm eradication reached 37.58 ± 5.02% for *C. acnes*, 25.80 ± 3.97% for *S. epidermidis*, and 1.60 ± 5.57% for *S. aureus*. At 2× MIC, treatment with the LCFS results in eradication rates of 25.31 ± 3.30, 26.89 ± 4.57% and 1.65 ± 2.11%, respectively. The highest eradication activity was observed against *C. acnes*, followed by *S. epidermidis*. These results indicate that the LCFS derived from *L. paracasei* T0901 eradicates biofilms established by acne-associated bacteria.

### 3.3. LCFS Derived from L. paracasei T0901 Exhibits Moderate Inhibition of Mushroom tyrosinase Activity

We evaluated the anti-tyrosinase activity of the LCFS derived from *L. paracasei* T0901 using a mushroom tyrosinase assay with L-DOPA as the substrate. Treatment with LCFS at concentrations of 5 and 10 mg/mL inhibited mushroom tyrosinase activity by 31.81 ± 6.29 and 34.85 ± 0.25%, respectively, compared to the untreated control. However, the positive control, kojic acid (1000 µg/mL), exhibited a significantly more potent inhibitory effect (83.83 ± 0.18%, *p* < 0.05) compared to the LCFS. These results indicate that the LCFS derived from *L. paracasei* T0901 exerts moderate tyrosinase inhibitory activity in a dose-dependent manner, suggesting the presence of bioactive compounds capable of interfering with the melanogenic enzyme cascade.

### 3.4. LCFS Derived from L. paracasei T0901 Does Not Affect B16F10 Cell Viability

We then determined the concentration of LCFS of *L. paracasei* T0901 that does not exert cytotoxic effects. To this end, we induced B16F10 cells with 1 µM of α-MSH and treated them with varying concentrations of LCFS (0.08–10.00 mg/mL). Induction with 1 µM α-MSH did not significantly affect cell viability compared to the untreated control group. Notably, treatment with the LCFS of *L. paracasei* T0901 exhibited no cytotoxicity toward B16F10 cells even at the highest concentration tested. We also quantified changes in the numbers of viable B16F10 cells treated with varying concentrations of the LCFS of *L. paracasei* T0901 (0.08–10.00 mg/mL) using the trypan blue dye exclusion method. We observed no significant differences in the viability of LCFS-treated cells compared to control cells. Therefore, we selected this concentration for subsequent experiments.

### 3.5. Effects of LCFS from L. paracasei T0901 on Melanin Content

**Figure 2:**
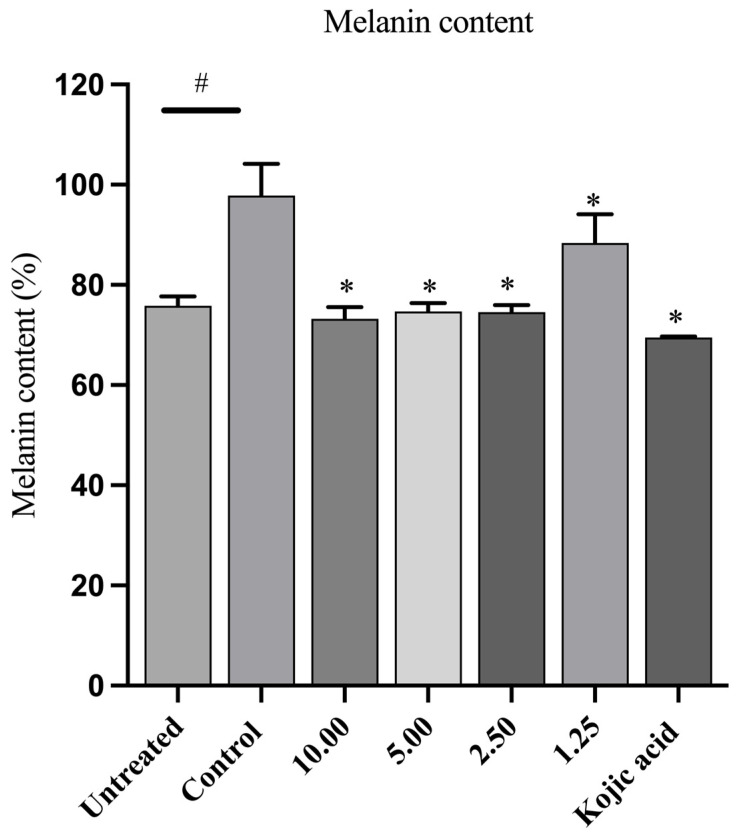
Effects of the LCFS of *Lacticaseibacillus paracasei* T0901 on melanin content. B16F10 cells were treated with 1 μM α-MSH in the presence of non-toxic concentrations of LCFS or kojic acid (1000 μg/mL; positive control) for 48 h. The control group consisted of cells treated with 1 μM α-MSH alone. Each bar represents the percentage relative to the control group. Data are presented as the mean ± standard error of the mean (SEM) from three independent experiments. # *p* < 0.05 compared to the untreated group; * *p* < 0.05 compared to the control group.

We evaluated the inhibitory effect of LCFS derived from *L. paracasei* T0901 on melanin production in α-MSH-stimulated B16F10 cells. We treated cells with various concentrations of LCFS (1.25–10.00 mg/mL) or kojic acid (500 µg/mL; positive control) for 48 h and then measured the melanin content. As illustrated in Figure 2, compared to control cells, α-MSH stimulation significantly (*p* < 0.05) increased melanin content. However, treatment with LCFS markedly reduced melanin synthesis in a dose-dependent manner. At a concentration of 10.00 mg/mL, LCFS decreased melanin content to 73.23 ± 2.36% of the control, whereas kojic acid reduced it to 69.52 ± 0.21%. These results indicate that the LCFS derived from *L. paracasei* T0901 exerts effective antimelanogenic effects in α-MSH-stimulated B16F10 cells.

### 3.6. LCFS from L. paracasei T0901 Inhibits Cellular Tyrosinase Activity in α-MSH-Stimulated B16F10 Cells

**Figure 3:**
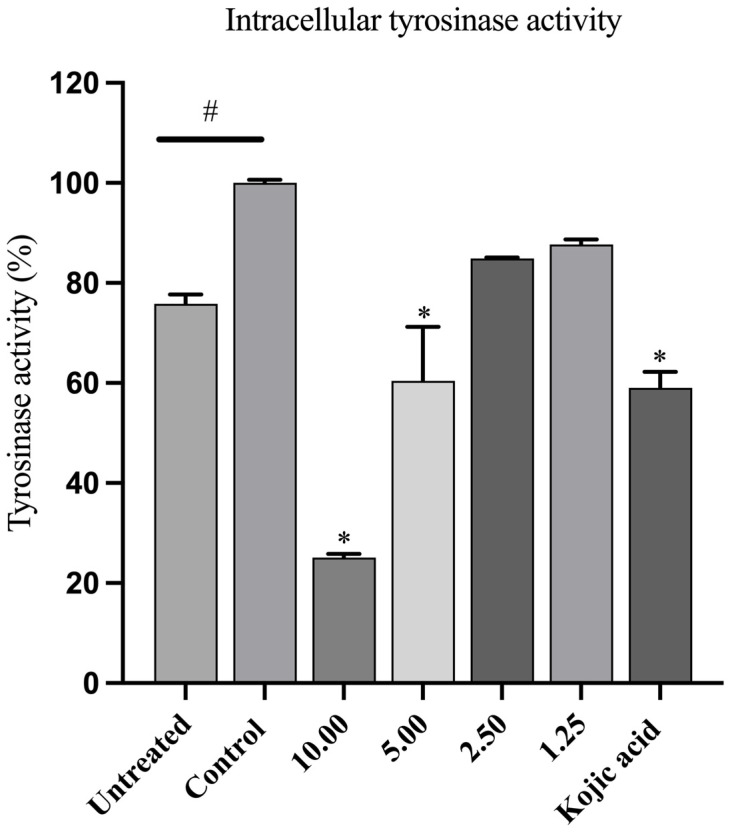
Effect of the LCFS of *Lacticaseibacillus paracasei* T0901 on intracellular tyrosinase activity in B16F10 cells. Cells were treated with 1 μM α-MSH in the presence of non-toxic concentrations of LCFS or kojic acid (1000 μg/mL; positive control) for 48 h. The control group consisted of cells treated with 1 μM α-MSH alone. Results are expressed as a percentage relative to the control group. Data are presented as the mean ± standard error of the mean (SEM) from three independent experiments. # *p* < 0.05 compared to the untreated group; * *p* < 0.05 compared to the control group.

We also investigated the effect of the LCFS derived from *L. paracasei* T0901 on intracellular tyrosinase activity in α-MSH-stimulated B16F10 cells. To this end, we induced cells with 1 µM α-MSH, followed by treatment with either LCFS (1.25–10.00 mg/mL) or kojic acid (500 µg/mL) for 48 h. α-MSH-induced B16F10 cells exhibited significantly increased tyrosinase activity compared to untreated cells (*p* < 0.05). In contrast, treatment with LCFS at 5.00 and 10.00 mg/mL significantly reduced intracellular tyrosinase activity compared with the control group (α-MSH only). Similarly, kojic acid, used as a positive control, significantly decreased tyrosinase activity (Figure 3). These findings suggest that the LCFS derived from *L. paracasei* T0901 inhibited intracellular tyrosinase activity in a dose-dependent manner.

### 3.7. WGS and Functional Gene Annotation

**Figure 4:**
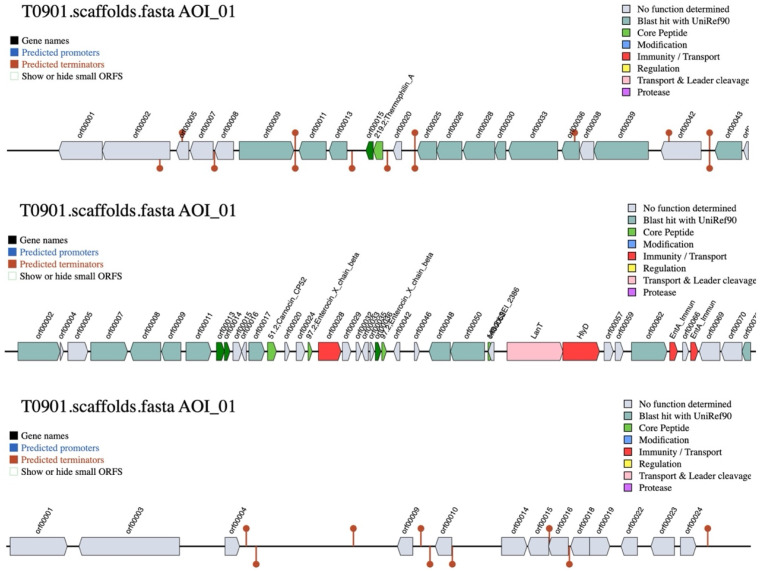
Gene cluster organization for bacteriocin biosynthesis in *Lacticaseibacillus paracasei* T0901. The cluster contains genes encoding core peptides, modification enzymes, transport, immunity, and regulatory proteins predicted using BAGEL analysis.

We conducted WGS of *L. paracasei* T0901 and identified several genes associated with antimicrobial activity and skin-beneficial properties. Specifically, we detected genes related to bacteriocin biosynthesis and immunity (Thermophilin_A, Carnocin_CP52, Enterocin_X_chain_beta, and LSEI_2386), supporting the potential inhibitory effects of the LCFS against acne-associated pathogens (Figure 4). In addition, the genome harbored stress tolerance genes, including cation-transporting ATPase and ABC superfamily ATPase components, which may enhance bacterial resilience under environmental stress conditions characteristic of the skin microbiome. Notably, we also identified genes putatively involved in tyrosinase-related and pigmentation-modulating processes, including multicopper oxidase (*mco*), gamma-glutamyl-gamma-aminobutyrate hydrolase (*fcbD*), and putative butyrate kinase (*buk*). These enzymes are associated with oxidative regulation, melanin metabolism, and short-chain fatty acid biosynthesis, collectively contributing to skin protection and pigmentation control. Furthermore, the genome encoded several oxidative stress defense genes, such as superoxide dismutase (*sodA*), NADH oxidase (*nox*), and thioredoxin reductase (*trxB*), which may enhance antioxidant capacity and protect skin cells from oxidative damage. Collectively, these genomic features suggest that *L. paracasei* T0901 possesses functional potential, including antimicrobial defense mechanisms and genes associated with skin protection, antioxidation, and tyrosinase modulation (Table S1).

### 3.8. Molecular Docking and Interaction Profile

**Figure 5:**
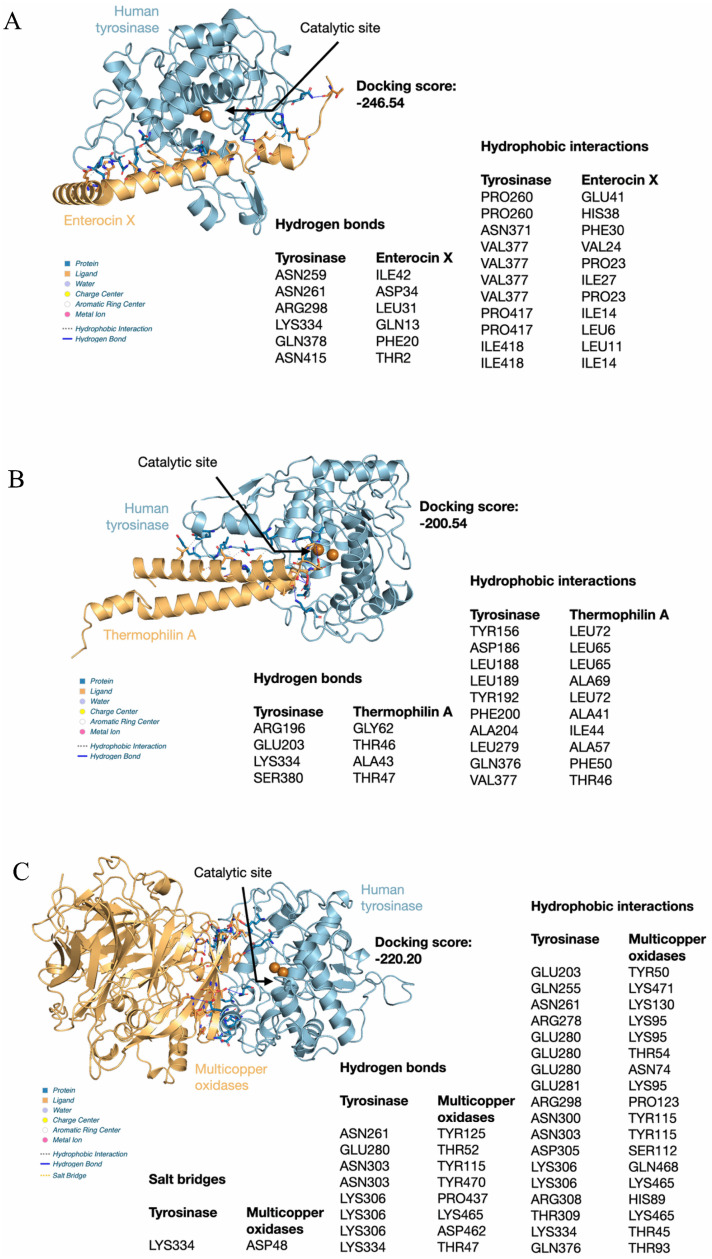
Predicted molecular docking complexes and non-covalent interactions between three selected microbial proteins and Human Tyrosinase. (**A**) Predicted structure, docking score, and interaction profile for the Enterocin X—Tyrosinase complex (Docking Score: −246.54). (**B**) Predicted structure, docking score, and interaction profile for the Thermophilin A—Tyrosinase complex (Docking Score: −200.54). (**C**) Predicted structure, docking score, and interaction profile for the Multicopper oxidase—Tyrosinase complex (Docking Score: −220.20). All panels highlight the close proximity of the microbial proteins (colored orange) to the Tyrosinase catalytic site (indicated by the arrow), and both spheres are copper atoms (colored brown) Tables in each panel detail the specific hydrogen bonds and hydrophobic interactions that stabilize the complex.

Seven microbial proteins and peptides were initially docked against human tyrosinase using the HDOCK Server. Following simulation, the results were filtered based on the docking score and the spatial orientation of the ligand relative to the tyrosinase catalytic site. Only three of the seven tested ligands, Enterocin X, Thermophilin A, and Multicopper oxidase yielded complexes that satisfied the stringent selection criteria (i.e., most favorable docking score among the top ten poses, combined with a suitable position near the catalytic site). The most favorable complexes were achieved with the following docking scores: Enterocin X (Figure 5A)—(−246.54); Multicopper oxidase (Figure 5C)—(−220.20); Thermophilin A (Figure 5B)—(−200.54). While these poses represent highly favorable binding scores, the visual analysis indicated that neither Enterocin X nor Multicopper oxidase directly blocked the catalytic site entrance. Instead, they were positioned close to the active site, suggesting a potential mechanism of action involving interference with the binding site of the natural substrate, tyrosine, rather than complete occlusion of the active pocket.

The selected complexes were subjected to detailed non-covalent interaction analysis using the Protein–Ligand Interaction Profiler (PLIP), focusing on the critical stabilizing forces. The most prominent and structurally crucial interactions across all complexes were the hydrogen bonds (H-bonds). The superior binding affinity (−246.54) of Enterocin X was stabilized by an extensive network of six H-bonds formed between Tyrosinase and the microbial protein. Key stabilizing H-bonds involved Tyrosinase residues ASN259, ASN261, ARG298, LYS334, GLN378, and ASN415. The Thermophilin A complex (−200.54) was anchored by four H-bonds, connecting Tyrosinase residues ARG196, GLU203, LYS334, and SER380. The Multicopper oxidase complex (−220.20) utilized eight H-bonds for structural anchorage. These bonds primarily involved Tyrosinase residues ASN261, GLU280, ASN303, LYS306, and LYS334. Specifically, three strong H-bonds were formed solely by LYS306, connecting to PRO437, LYS465, and ASP462 of the Multicopper oxidase. Crucially, comparative analysis revealed that LYS334 on Human Tyrosinase served as a key common anchoring residue for all three microbial proteins (Enterocin X, Thermophilin A, and Multicopper oxidase), underscoring its importance in their binding mechanism. Additionally, ASN261 was critical in stabilizing the two highest-scoring complexes, Enterocin X and Multicopper oxidase. Furthermore, the Multicopper oxidase complex was uniquely reinforced by a cationic interaction (salt bridge) formed between the positively charged LYS334 on Tyrosinase and the negatively charged ASP48 on the Multicopper oxidase, contributing to its substantial stability.

## 4. Discussion

Probiotics are receiving considerable attention for their health-promoting properties, including their ability to regulate intestinal microbiota. They also exhibit a range of biological activities, including reduction in oxidative damage, suppression of pigment formation, maintenance of skin structural integrity, attenuation of age-related cellular decline, modulation of inflammatory responses, inhibition of tumor cell proliferation, and alleviation of allergic reactions [11,42]. In recent years, LAB, particularly strains belonging to the *Lacticaseibacillus* genus, have demonstrated considerable potential. These strains produce diverse bioactive compounds, including bacteriocins, short-chain fatty acids, and organic acid metabolites, which exhibit valuable applications in therapeutic and cosmetic formulations [11]. Fermented foods are important natural sources of probiotics, produced through microbial metabolism during the fermentation process. Traditional products such as yogurt, kimchi, sauerkraut, and kombucha contain various LAB, contributing to flavor, preservation, and potential probiotic effects [43]. In tropical regions, fermented plant-based substrates harbor rich yet unexplored microbial ecosystems. For example, fermented palm sap, a traditional beverage widely consumed in Southeast Asia, hosts diverse LAB species, such as *Lacticaseibacillus*, adapted to high-sugar and acidic conditions [29]. Notably, these microorganisms can produce bioactive metabolites with antimicrobial, antioxidant, and anti-inflammatory properties [19]. In the present study, we investigated the biological properties of the LCFS of *L. paracasei* T0901 isolated from fermented palm sap.

*Lacticaseibacillus paracasei* is widely recognized for its ability to promote intestinal health, enhance immune responses, and modulate host–microbe interactions [44]. Beyond gastrointestinal benefits, *L. paracasei* exerts protective effects on the skin, with its CFS demonstrating antimicrobial activity against acne-associated bacteria and inhibitory effects on melanogenesis in skin cells [27,45]. Consistent with these reported effects, in the present study, we demonstrated that the LCFS of *L. paracasei* T0901 exhibited both antiacne and antimelanogenic activities.

Acne vulgaris is a common dermatological condition with a multifactorial pathogenesis, including excess sebum secretion, abnormal follicular keratinization, bacterial colonization, inflammatory cascade activation, and pathogenic microbial invasion [1,2]. For example, *C. acnes* proliferates and releases lipases, proteases, and hyaluronidases that degrade sebum triglycerides and disrupt follicular integrity [46]. This process triggers Toll-like receptor-mediated signaling in keratinocytes and sebocytes, promoting the production of proinflammatory cytokines such as interleukin (IL)-1β, IL-6, and tumor necrosis factor-α [46]. In addition, *S. aureus* and *S. epidermidis* contribute to acne severity. Specifically, *S. aureus*, a facultative anaerobe commonly present on human skin, can invade damaged follicles, produce toxins and proteases, and exacerbate inflammation and pustule formation. Moreover, *S. epidermidis* can become opportunistic under dysbiotic conditions, forming biofilms and producing extracellular polymeric substances that enhance the tolerance of *C. acnes* and *S. aureus* to antimicrobial agents [46,47]. In this study, the LCFS derived from *L. paracasei* T0901 exhibited significant antimicrobial properties against *C. acnes*, *S. aureus*, and *S. epidermidis*. WGS analysis identified genes associated with bacteriocin biosynthesis, including Thermophilin_A, Carnocin_CP52, Enterocin_X_chain_beta, and LSEI_2386, which likely contribute to these antimicrobial effects. Thermophilin A forms an ionophoric pore complex through the interaction of ThmA and ThmB peptides, independent of a receptor [48]. Carnocin CP52 contains a conserved alpha-helix and amphipathic YGNGV(X)C(X)4C motif that permeabilizes membranes [49]. Enterocin X chain beta form pores through its amphiphilic helical structure in combination with two complementary peptides [50]. Furthermore, SEI_2386 causes leakage of K^+^ and inorganic phosphate while decreasing membrane potential [51]. All four bacteriocins insert into target cell membranes via amphipathic structures, create pores, compromise membrane integrity, and induce bactericidal death through cellular leakage [52]. Notably, *L. plantarum*-GMNL6 can inhibit *S. aureus* and *C. acnes* [25], *L. plantarum* APsulloc 331261 and APsulloc 331266 are effective against five skin pathogenic strains [26], and *L. paracasei* LPH01 effectively inhibits *P. acnes* [53]. Consistent with these findings, in our study, the LCFS of *L. paracasei* T0901 inhibited biofilm formation and partially eradicated preformed biofilms of *C. acnes*, *S. aureus*, and *S. epidermidis*.

Melanin plays a key role in protecting skin from UV-induced oxidative stress. However, its excessive accumulation can cause hyperpigmentation disorders, such as melasma, freckles, and age spots, posing major cosmetic concerns [3,4]. Skin pigmentation occurs when UV radiation stimulates keratinocytes to secrete α-MSH, which binds to MC1R on melanocytes and activates downstream signaling cascades that promote melanogenesis [3,4]. Moreover, α-MSH stimulation leads to increased expression and activity of tyrosinase, which catalyzes the rate-limiting steps of melanogenesis. Accordingly, tyrosinase has become a major therapeutic target in pigmentation research [54]. In this study, the LCFS derived from *L. paracasei* T0901 significantly reduced intracellular melanin content and tyrosinase activity in α-MSH-stimulated B16F10 cells without inducing cytotoxicity. These results are consistent with previous reports demonstrating that *L. gasseri* BNR17 and *L. fermentum* JNU532 suppress melanin synthesis by downregulating tyrosinase and oxidative stress in melanocytes [27,28]. Moreover, WGS revealed that *L. paracasei* T0901 harbors genes associated with pigmentation control and antioxidant defense, including *mco*, *fcbD*, and *buk*. Notably, *mco* may interfere with tyrosinase activity, as this copper-dependent enzyme requires dinuclear copper ions for melanin synthesis. Moreover, *fcbD*, which is involved in GABA metabolism, may contribute to the antimelanogenic effects through GABA-mediated downregulation of MITF and tyrosinase expression through inhibition of the cAMP/CREB pathway. Finally, *buk* encodes the terminal enzyme in butyrate biosynthesis, indicating SCFA-producing potential that may mitigate inflammation and oxidative stress, indirectly modulating melanogenesis [55]. Additional antioxidant-related genes (*sodA*, *nox*, and *trxB*) suggest an intrinsic ability to neutralize ROS, supporting cell protection [45,56]. These findings were further supported by molecular docking. Specifically, LCFS-derived metabolites exhibited binding affinities toward tyrosinase, MITF, and GPCRs, with low binding free energies and inhibition constants, indicating stable inhibitory interactions. Notably, the identified hydrogen bonding and π–π stacking with catalytic residues suggest a competitive inhibition mechanism similar to that of known tyrosinase inhibitors

In summary, this study demonstrated that *L. paracasei* T0901 exhibits multifunctional bioactivities, including antimicrobial, antibiofilm, antioxidant, and antimelanogenic effects, making it an attractive postbiotic candidate for functional skincare applications. It demonstrated a favorable safety profile and efficacy, highlighting its potential as a natural alternative to conventional whitening and antiacne agents, with promising applications in cosmetics and therapeutics.

## 5. Conclusions

The LCFS of *L. paracasei* T0901 isolated from fermented palm sap exhibited strong antimicrobial, antibiofilm, and antimelanogenic activities without inducing cytotoxicity. It effectively inhibited acne-associated pathogens and reduced melanin synthesis and tyrosinase activity in α-MSH-stimulated B16F10 cells. Moreover, WGS revealed the presence of genes related to antioxidant defense and metabolite biosynthesis, supporting its multifunctional skin benefits. Molecular docking confirmed strong interactions between LCFS-derived metabolites and melanogenic targets, suggesting modulation of tyrosinase and MITF pathways. Overall, *L. paracasei* T0901 is a promising probiotic source of safe, natural postbiotic ingredients for antiacne and skin-brightening cosmetic formulations. Future studies should include standard antibacterial reference agents as control and should focus on comprehensive metabolite identification through liquid chromatography–tandem mass spectrometry analysis, detailed elucidation of the signaling pathways involved in the suppression of melanogenesis, and clinical evaluation of topical formulations incorporating LCFS to validate its efficacy and safety in humans.

## Figures and Tables

**Table 1:** Minimal inhibitory concentration (MIC) and minimal bactericidal concentration (MBC) of LCFS from *Lactobacillus* against the three pathogens.

Isolates	Antimicrobial Activity (mg/mL)					
	*Cutibacterium acnes* ATCC 6919		*Staphylococcus epidermidis* DMST 15548		*Staphylococcus aureus* DMST 4745	
	MIC	MBC	MIC	MBC	MIC	MBC
LCFS from *Lacticaseibacillus paracasei* T0901	25	>50	25	>50	25	>50

This test was performed in triplicate.

## Data Availability

The original contributions presented in this study are included in the article/Supplementary Materials. Further inquiries can be directed to the corresponding author.

## Supplementary Materials

The following supporting information can be downloaded at: https://www.mdpi.com/article/10.3390/life15121866/s1, Table S1. Genes summary of *Lacticaseibacillus paracasei* T0901.

## References

[1] Deng Y., Wang F., He L. (2024). Skin Barrier Dysfunction in Acne Vulgaris: Pathogenesis and Therapeutic Approaches. Med. Sci. Monit..

[2] Knutsen-Larson S., Dawson A.L., Dunnick C.A., Dellavalle R.P. (2012). Acne vulgaris: Pathogenesis, treatment, and needs assessment. Dermatol. Clin..

[3] Karkoszka M., Rok J., Wrześniok D. (2024). Melanin Biopolymers in Pharmacology and Medicine-Skin Pigmentation Disorders, Implications for Drug Action, Adverse Effects and Therapy. Pharmaceuticals.

[4] Brenner M., Hearing V.J. (2008). The protective role of melanin against UV damage in human skin. Photochem. Photobiol..

[5] Yamaguchi Y., Hearing V. (2009). Physiological factors that regulate skin pigmentation. BioFactors.

[6] Guermazi D., Saliba E. (2025). The Genetics and Evolution of Human Pigmentation. Biology.

[7] Wohlrab J., Kreft D. (2014). Niacinamide—Mechanisms of action and its topical use in dermatology. Skin Pharmacol. Physiol..

[8] Saeedi M., Eslamifar M., Khezri K. (2019). Kojic acid applications in cosmetic and pharmaceutical preparations. Biomed. Pharmacother..

[9] Pillaiyar T., Manickam M., Namasivayam V. (2017). Skin whitening agents: Medicinal chemistry perspective of tyrosinase inhibitors. J. Enzym. Inhib. Med. Chem..

[10] Neto C., Nascimento P., Silveira V., Mattos A., Bertol C. (2022). Natural Sources of Melanogenic Inhibitors: A Systematic Review. Int. J. Cosmet. Sci..

[11] Puebla-Barragan S., Reid G. (2021). Probiotics in Cosmetic and Personal Care Products: Trends and Challenges. Molecules.

[12] Zeng M., Li Y., Cheng J., Wang J., Liu Q. (2025). Prebiotic Oligosaccharides in Skin Health: Benefits, Mechanisms, and Cosmetic Applications. Antioxidants.

[13] Son E. (2025). The potential of probiotics derived from functional foods for skin health. Czech J. Food Sci..

[14] Future Market Insights. Probiotic Skincare Ingredients Market. 12 October 2025. https://www.futuremarketinsights.com/reports/probiotic-skincare-ingredients-market.

[15] Hill C., Guarner F., Reid G., Gibson G.R., Merenstein D.J., Pot B., Morelli L., Canani R.B., Flint H.J., Salminen S. (2014). Expert consensus document. The International Scientific Association for Probiotics and Prebiotics consensus statement on the scope and appropriate use of the term probiotic. Nat. Rev. Gastroenterol. Hepatol..

[16] Suez J., Zmora N., Segal E., Elinav E. (2019). The pros, cons, and many unknowns of probiotics. Nat. Med..

[17] Li Z., Li P., Xu Y., Yan C., Ma X., Wang H., Cheng H., Zeng J., Li T., Li X. (2025). Efficacy of a Postbiotic Formulation Combined With Microneedling for Mild-to-Moderate Acne: A Self-Control Study. J. Cosmet. Dermatol..

[18] Sornsenee P., Chatatikun M., Mitsuwan W., Kongpol K., Kooltheat N., Sohbenalee S., Pruksaphanrat S., Mudpan A., Romyasamit C. (2021). Lyophilized cell-free supernatants of *Lactobacillus* isolates exhibited antibiofilm, antioxidant, and reduces nitric oxide activity in lipopolysaccharide-stimulated RAW 264.7 cells. PeerJ.

[19] Sornsenee P., Kooltheat N., Wongprot D., Suksabay P., Nam T.G., Permpoon U., Saengsuwan P., Romyasamit C. (2025). Antibacterial, Antioxidant, and Anti-inflammatory Activities of *Lacticaseibacillus paracasei* Lysates Isolated from Fermented Palm Sap. Probiotics Antimicrob Proteins.

[20] Machado P., Ribeiro F.N., Giublin F.C.W., Mieres N.G., Tonin F.S., Pontarolo R., Sari M.H.M., Lazo R.E.L., Ferreira L.M. (2025). Next-Generation Wound Care: A Scoping Review on Probiotic, Prebiotic, Synbiotic, and Postbiotic Cutaneous Formulations. Pharmaceuticals.

[21] Bustamante M., Oomah B.D., Oliveira W.P., Burgos-Díaz C., Rubilar M., Shene C. (2020). Probiotics and prebiotics potential for the care of skin, female urogenital tract, and respiratory tract. Folia Microbiol..

[22] Knackstedt R., Knackstedt T., Gatherwright J. (2020). The role of topical probiotics in skin conditions: A systematic review of animal and human studies and implications for future therapies. Exp. Dermatol..

[23] Eguren C., Navarro-Blasco A., Corral-Forteza M., Reolid-Pérez A., Setó-Torrent N., García-Navarro A., Prieto-Merino D., Núñez-Delegido E., Sánchez-Pellicer P., Navarro-López V. (2024). A Randomized Clinical Trial to Evaluate the Efficacy of an Oral Probiotic in Acne Vulgaris. Acta Derm.-Venereol..

[24] Li Y., Hu X., Dong G., Wang X., Liu T. (2024). Acne treatment: Research progress and new perspectives. Front. Med..

[25] Tsai W.H., Chou C.H., Chiang Y.J., Lin C.G., Lee C.H. (2021). Regulatory effects of *Lactobacillus plantarum*-GMNL6 on human skin health by improving skin microbiome. Int. J. Med. Sci..

[26] Chae M., Kim B.J., Na J., Kim S.-Y., Lee J.O., Kim Y.-J., Lee E., Cho D., Roh J., Kim W. (2021). Antimicrobial activity of *Lactiplantibacillus plantarum* APsulloc 331261 and APsulloc 331266 against pathogenic skin microbiota. FBE.

[27] Meng Z., Oh S. (2021). Antioxidant and Antimelanogenic Activities of Kimchi-Derived *Limosilactobacillus fermentum* JNU532 in B16F10 Melanoma Cells. J. Microbiol. Biotechnol..

[28] Lee S., Park H.O., Yoo W. (2022). Anti-Melanogenic and Antioxidant Effects of Cell-Free Supernatant from *Lactobacillus gasseri* BNR17. Microorganisms.

[29] Sornsenee P., Singkhamanan K., Sangkhathat S., Saengsuwan P., Romyasamit C. (2021). Probiotic Properties of *Lactobacillus* Species Isolated from Fermented Palm Sap in Thailand. Probiotics Antimicrob Proteins.

[30] Romyasamit C., Thatrimontrichai A., Aroonkesorn A., Chanket W., Ingviya N., Saengsuwan P., Singkhamanan K. (2020). *Enterococcus faecalis* Isolated From Infant Feces Inhibits Toxigenic *Clostridioides* (*Clostridium*) *difficile*. Front. Pediatr..

[31] Li M., Liu Z., He J., Jiang J., Shang D., Dong W. (2025). Trp-containing Peptides with Therapeutic Potential for *Cutibacterium acnes* Infection. Probiotics Antimicrob. Proteins.

[32] Yang K.M., Kim J.-S., Kim H.-S., Kim Y.-Y., Oh J.-K., Jung H.-W., Park D.-S., Bae K.-H. (2021). *Lactobacillus reuteri* AN417 cell-free culture supernatant as a novel antibacterial agent targeting oral pathogenic bacteria. Sci. Rep..

[33] Perumal S., Mahmud R. (2013). Chemical analysis, inhibition of biofilm formation and biofilm eradication potential of *Euphorbia hirta* L. against clinical isolates and standard strains. BMC Complement. Altern. Med..

[34] Lee J.E., An B.J., Jo C., Min B., Paik H.D., Ahn D.U. (2023). The elastase and melanogenesis inhibitory and anti-inflammatory activities of phosvitin phosphopeptides produced using high-temperature and mild-pressure (HTMP) pretreatment and enzyme hydrolysis combinations. Poult. Sci..

[35] Kooltheat N., Tedasen A., Yamasaki K., Chatatikun M. (2023). Melanogenesis Inhibitory Activity, Chemical Components and Molecular Docking Studies of *Prunus cerasoides* Buch.-Ham. D. Don. Flowers. J. Evid.-Based Integr. Med..

[36] Lee H.-W., Lee Y.-R., Park K.-M., Lee N.-K., Paik H.-D. (2024). Antimelanogenic and Antioxidant Effects of Postbioics of *Lactobacillus* Strains in α-MSH-Induced B16F10 Melanoma Cells via CREB/MITF and MAPKs Signaling Pathway. J. Microbiol. Biotechnol..

[37] van Heel A.J., de Jong A., Song C.X., Viel J.H., Kok J., Kuipers O.P. (2018). BAGEL4: A user-friendly web server to thoroughly mine RiPPs and bacteriocins. Nucleic Acids Res..

[38] Varadi M., Anyango S., Deshpande M., Nair S., Natassia C., Yordanova G., Yuan D., Stroe O., Wood G., Laydon A. (2022). AlphaFold Protein Structure Database: Massively expanding the structural coverage of protein-sequence space with high-accuracy models. Nucleic Acids Res..

[39] Varadi M., Bertoni D., Magana P., Paramval U., Pidruchna I., Radhakrishnan M., Tsenkov M., Nair S., Mirdita M., Yeo J. (2024). AlphaFold Protein Structure Database in 2024: Providing structure coverage for over 214 million protein sequences. Nucleic Acids Res..

[40] Yan Y., Tao H., He J., Huang S.-Y. (2020). The HDOCK server for integrated protein–protein docking. Nat. Protoc..

[41] Salentin S., Schreiber S., Haupt V.J., Adasme M.F., Schroeder M. (2015). PLIP: Fully automated protein-ligand interaction profiler. Nucleic Acids Res..

[42] Yadav M.K., Kumari I., Singh B., Sharma K.K., Tiwari S.K. (2022). Probiotics, prebiotics and synbiotics: Safe options for next-generation therapeutics. Appl. Microbiol. Biotechnol..

[43] Kok C.R., Hutkins R. (2018). Yogurt and other fermented foods as sources of health-promoting bacteria. Nutr. Rev..

[44] Kullar R., Goldstein E.J.C., Johnson S., McFarland L.V. (2023). *Lactobacillus* Bacteremia and Probiotics: A Review. Microorganisms.

[45] Huang H.C., Lee I.J., Huang C., Chang T.M. (2020). Lactic Acid Bacteria and Lactic Acid for Skin Health and Melanogenesis Inhibition. Curr. Pharm. Biotechnol..

[46] Scott E., Burkhart C. (2025). A Review of the Role of C. Acnes and its Biofilm in Dandruff Pathogenesis. J. Drugs Dermatol..

[47] Kreouzi M., Theodorakis N., Nikolaou M., Feretzakis G., Anastasiou A., Kalodanis K., Sakagianni A. (2025). Skin Microbiota: Mediator of Interactions Between Metabolic Disorders and Cutaneous Health and Disease. Microorganisms.

[48] Salini F., Iacumin L., Comi G., Dicks L.M.T. (2023). Thermophilin 13: In Silico Analysis Provides New Insight in Genes Involved in Bacteriocin Production. Microorganisms..

[49] Leisner J.J., Laursen B.G., Prévost H., Drider D., Dalgaard P. (2007). *Carnobacterium*: Positive and negative effects in the environment and in foods. FEMS Microbiol. Rev..

[50] Wu Y., Pang X., Wu Y., Liu X., Zhang X. (2022). Enterocins: Classification, Synthesis, Antibacterial Mechanisms and Food Applications. Molecules.

[51] Du R., Ping W., Ge J. (2022). Purification, characterization and mechanism of action of enterocin HDX-2, a novel class IIa bacteriocin produced by *Enterococcus faecium* HDX-2. LWT.

[52] Sornsenee P., Surachat K., Kang D.-K., Mendoza R., Romyasamit C. (2024). Probiotic Insights from the Genomic Exploration of *Lacticaseibacillus paracasei* Strains Isolated from Fermented Palm Sap. Foods.

[53] Xu J., Chen X., Song J., Wang C., Xu W., Tan H., Suo H. (2024). Antibacterial activity and mechanism of cell-free supernatants of *Lacticaseibacillus paracasei* against Propionibacterium acnes. Microb. Pathog..

[54] D’Mello S.A., Finlay G.J., Baguley B.C., Askarian-Amiri M.E. (2016). Signaling pathways in melanogenesis. Int. J. Mol. Sci..

[55] Xiao X., Hu X., Yao J., Cao W., Zou Z., Wang L., Qin H., Zhong D., Li Y., Xue P. (2022). The role of short-chain fatty acids in inflammatory skin diseases. Front. Microbiol..

[56] Di Chiano M., Rocchetti M.T., Spano G., Russo P., Allegretta C., Milior G., Gadaleta R.M., Sallustio F., Pontrelli P., Gesualdo L. (2024). Lactobacilli Cell-Free Supernatants Modulate Inflammation and Oxidative Stress in Human Microglia via NRF2-SOD1 Signaling. Cell. Mol. Neurobiol..

