# Supplementary material for: Lyophilized Cell-Free Supernatants of Lacticaseibacillus paracasei T0901 Isolated from Fermented Palm Sap Exhibit Antiacne and Antimelanogenic Activities in B16F10 Melanoma Cells
Source: Life (Basel). 2025 Dec 5;15(12):1866. doi: 10.3390/life15121866 (PMC12733966; doi:10.3390/life15121866)
Supplement: Supplementary file 1 [file life-15-01866-s001.zip › life-4016747-supplementary.pdf]

**Supplementary Table S1.** Genes summary of *Lacticaseibacillus paracasei* T0901

| Category | gene_name | seqid | start | end | strand |
|---|---|---|---|---|---|
| Adhesion & surface proteins | Cell surface protein | contig_5 | 47212 | 47583 | + |
| Adhesion & surface proteins | Cell surface protein | contig_5 | 47658 | 49607 | + |
| Adhesion & surface proteins | Cell surface protein | contig_6 | 87532 | 88440 | + |
| Adhesion & surface proteins | Cell surface protein | contig_7 | 92967 | 94562 | - |
| Bacteriocins | Class IIb bacteriocin%2C lactobin A/cerein 7B family | contig_1 | 53097 | 53312 | + |
| Bacteriocins | Bacteriocin immunity protein | contig_1 | 54742 | 55077 | + |
| Bacteriocins | AbpD bacteriocin export accessory protein | contig_1 | 65989 | 67368 | + |
| Bacteriocins | Bacteriocin immunity protein | contig_1 | 70039 | 70335 | + |
| Bacteriocins | Bacteriocin immunity protein | contig_1 | 70528 | 70812 | + |
| Bacteriocins | Prebacteriocin | contig_1 | 70836 | 71114 | + |
| Bacteriocins | Bacteriocin | contig_4 | 211903 | 212085 | - |
| Bacteriocins | Bacteriocin-type signal sequence | contig_4 | 212136 | 212315 | - |
| Bacteriocins | Bacteriocin | contig_10 | 15819 | 15983 | - |
| Bacteriocins | Class IIb bacteriocin%2C lactobin A/cerein 7B family | contig_10 | 16186 | 16374 | - |
| Glutathione metabolism | Glutathione reductase | contig_5 | 126427 | 127752 | + |
| Glutathione metabolism | Glutathione peroxidase | contig_12 | 75037 | 75510 | - |
| Stress tolerance | ATPase component of an ABC superfamily oligopeptide transporter | contig_1 | 1295 | 2245 | + |
| Stress tolerance | Cation-transporting ATPase | contig_1 | 13407 | 15662 | - |
| Stress tolerance | Cation-ATPase-N domain-containing protein | contig_1 | 42140 | 44857 | - |
| Stress tolerance | ABC-type sugar transport system%2C ATPase component | contig_1 | 115649 | 116425 | + |
| Stress tolerance | Magnesium-transporting ATPase%2C P-type 1 | contig_1 | 155846 | 158476 | + |
| Stress tolerance | Chaperonin GroEL | contig_1 | 202308 | 203942 | + |
| Stress tolerance | ATP-dependent protease ATPase subunit HslU | contig_2 | 45621 | 47030 | - |

| Category | gene_name | seqid | start | end | strand |
|---|---|---|---|---|---|
| Stress tolerance | ATPase component of an ABC superfamily transporter | contig_2 | 63470 | 65362 | - |
| Stress tolerance | Chaperone protein ClpB | contig_2 | 93803 | 96409 | - |
| Stress tolerance | ABC-type polar amino acid transport system%2C ATPase component | contig_2 | 108704 | 109450 | - |
| Stress tolerance | ATP-dependent Clp protease ATP-binding subunit ClpX | contig_2 | 112365 | 113615 | - |
| Stress tolerance | ATPase | contig_2 | 144620 | 145435 | - |
| Stress tolerance | ABC-type polar amino acid transport system%2C ATPase component | contig_2 | 189801 | 190439 | - |
| Stress tolerance | Chaperone protein DnaK | contig_3 | 20103 | 21977 | - |
| Stress tolerance | Lead%2C cadmium%2C zinc and mercury transporting ATPase / Copper-translocating P-type ATPase | contig_3 | 197156 | 199402 | - |
| Stress tolerance | CopAB ATPases metal-fist type repressor | contig_3 | 199395 | 199835 | - |
| Stress tolerance | ATP-dependent Clp protease ATP-binding subunit ClpA | contig_3 | 216906 | 219002 | + |
| Stress tolerance | ATPase component of an ABC superfamily sugar transporter | contig_4 | 48643 | 49761 | - |
| Stress tolerance | Cadmium-translocating P-type ATPase | contig_4 | 84607 | 86454 | - |
| Stress tolerance | Putative ATPase | contig_4 | 138151 | 139086 | + |
| Stress tolerance | ATPase/permease component of an ABC superfamily multidrug transporter | contig_5 | 155118 | 156941 | - |
| Stress tolerance | HATPase-c-5 domain-containing protein | contig_5 | 156995 | 157906 | - |
| Stress tolerance | Cation transporting P-type ATPase | contig_6 | 110751 | 113414 | + |
| Stress tolerance | ATP-dependent Clp protease ATP-binding subunit | contig_7 | 2364 | 4514 | + |
| Stress tolerance | ATPase/permease component of an ABC superfamily multidrug transporter | contig_8 | 9273 | 10853 | + |

| Category | gene_name | seqid | start | end | strand |
|---|---|---|---|---|---|
| Stress tolerance | ATPase domain-containing PTS system mannose/fructose/N-acetylgalactosamine-specific IID component | contig_8 | 13355 | 14029 | - |
| Stress tolerance | Heavy metal transporting ATPase | contig_8 | 70939 | 72798 | - |
| Stress tolerance | Lead%2C cadmium%2C zinc and mercury transporting ATPase / Copper-translocating P-type ATPase | contig_8 | 112179 | 114035 | - |
| Stress tolerance | ATPase/permease component of an ABC superfamily multidrug transporter | contig_8 | 126460 | 128052 | - |
| Stress tolerance | Cation-ATPase-N domain-containing protein | contig_9 | 95522 | 98314 | + |
| Stress tolerance | Energizing module ATPase component of thiamin-regulated hydroxymethylpyrimidine ECF transporter | contig_10 | 57573 | 58916 | + |
| Stress tolerance | ATP-dependent Clp protease ATP-binding subunit ClpC | contig_15 | 1493 | 4000 | + |
| Stress tolerance | Cation transporter E1-E2 family ATPase | contig_16 | 16874 | 19534 | - |
| Stress tolerance | Ribosome-binding ATPase YchF | contig_18 | 14740 | 15762 | + |
| Stress tolerance | ATPase component of an ABC superfamily transporter | contig_18 | 47646 | 49193 | + |
| Stress tolerance | Abc-type multidrug transport system%2C atpase and permease component | contig_19 | 35446 | 37257 | - |
| Stress tolerance | Chromosome-partitioning ATPase Soj | contig_27 | 2696 | 3508 | - |
| Stress tolerance | ATP-dependent Clp protease proteolytic subunit | contig_30 | 8951 | 9679 | - |
| Stress tolerance | Phosphate-transporting ATPase | contig_33 | 11201 | 11818 | - |
| Stress tolerance | ATP-dependent Clp protease proteolytic subunit | contig_35 | 9493 | 10083 | - |
| Stress tolerance | Cadmium-translocating P-type ATPase | contig_47 | 861 | 2687 | + |

| Category | gene_name | seqid | start | end | strand |
|---|---|---|---|---|---|
| Stress tolerance | ATP-dependent Clp protease proteolytic subunit | contig_49 | 2793 | 3371 | - |
| Tyrosinase-Related_Genes | Multicopper oxidase mco | contig_1 | 38379 | 39908 | + |
| Tyrosinase-Related_Genes | putative butyrate kinase | contig_2 | 860 | 1981 | + |
| Tyrosinase-Related_Genes | Gamma-glutamyl-gamma-aminobutyrate hydrolase family protein | contig_21 | 24601 | 25353 | + |
| Vitamin biosynthesis | Foldase protein PrsA | contig_1 | 167238 | 168137 | - |
| Vitamin biosynthesis | Folate transporter FolT | contig_1 | 190204 | 190722 | + |
| Vitamin biosynthesis | zinc-ribbon-2 domain-containing protein | contig_1 | 304523 | 305602 | - |
| Vitamin biosynthesis | zinc-ribbon-2 domain-containing protein | contig_1 | 310592 | 311689 | + |
| Vitamin biosynthesis | zinc-ribbon-2 domain-containing protein | contig_1 | 312919 | 313707 | + |
| Vitamin biosynthesis | Methylenetetrahydrofolate--tRNA-(uracil-5-)-methyltransferase TrmFO | contig_2 | 48867 | 50186 | - |
| Vitamin biosynthesis | Dihydrofolate reductase | contig_2 | 61872 | 62363 | - |
| Vitamin biosynthesis | Riboflavin transporter | contig_2 | 77332 | 77910 | - |
| Vitamin biosynthesis | Iron-sulfur cluster assembly scaffold protein | contig_2 | 257291 | 257737 | - |
| Vitamin biosynthesis | Riboflavin biosynthesis protein | contig_3 | 25130 | 26077 | - |
| Vitamin biosynthesis | Bifunctional protein FolD | contig_3 | 98641 | 99492 | - |
| Vitamin biosynthesis | Foldase protein PrsA | contig_3 | 175929 | 176831 | + |
| Vitamin biosynthesis | MBL fold metallo-hydrolase | contig_6 | 39708 | 39869 | - |
| Vitamin biosynthesis | Alpha beta fold family hydrolase | contig_6 | 67564 | 68496 | - |

| Category | gene_name | seqid | start | end | strand |
|---|---|---|---|---|---|
| Vitamin biosynthesis | 5-formyltetrahydrofolate cyclo-ligase | contig_6 | 86877 | 87437 | + |
| Vitamin biosynthesis | Zinc-ribbon-2 domain-containing protein | contig_9 | 46336 | 47529 | + |
| Vitamin biosynthesis | Methylenetetrahydrofolate reductase | contig_17 | 5782 | 6654 | - |
| Vitamin biosynthesis | Thioredoxin-like-fold domain-containing protein | contig_20 | 187 | 495 | + |
| Vitamin biosynthesis | Formate--tetrahydrofolate ligase | contig_23 | 16846 | 18519 | + |
| Other/Unclassified | Glycosyl transferase family 8 domain-containing protein | contig_9 | 73980 | 74936 | - |
